# Total adiponectin is associated with incident cardiovascular and renal events in treated hypertensive patients: subanalysis of the ATTEMPT-CVD randomized trial

**DOI:** 10.1038/s41598-019-52977-x

**Published:** 2019-11-12

**Authors:** Shokei Kim-Mitsuyama, Hirofumi Soejima, Osamu Yasuda, Koichi Node, Hideaki Jinnouchi, Eiichiro Yamamoto, Taiji Sekigami, Hisao Ogawa, Kunihiko Matsui

**Affiliations:** 1 Department of Pharmacology and Molecular Therapeutics, Graduate School of Medical Sciences, Kumamoto University, Kumamoto, Japan; 2 Department of Cardiovascular Medicine, Graduate School of Medical Sciences, Kumamoto University, Kumamoto, Japan; 3 Health Care Center, Kumamoto University, Kumamoto, Japan; 4 Department of Sports and Life Sciences, National Institute of Fitness and Sports in Kanoya, Kanoya, Japan; 5 Department of Cardiovascular Medicine, Saga University, Saga, Japan; 6 Diabetes Care Center, Jinnouchi Clinic, Kumamoto, Japan; 7 Division of Internal Medicine & Diabetes and Endocrine, Sekigami Clinic, Yatsushiro, Japan; 8 National Cerebral and Cardiovascular Center, Suita, Japan; 9 Department of General and Community Medicine, Kumamoto University Hospital, Kumamoto, Japan

**Keywords:** Predictive markers, Hypertension

## Abstract

The predictive value of serum adiponectin for hypertensive cardiovascular outcomes is unknown. This study was performed to investigate the association of adiponectin with incident cardiovascular and renal events (CV events) in hypertensive patients. We performed post-hoc analysis on 1,228 hypertensive patients enrolled in the ATTEMPT-CVD study, a prospective randomized study comparing the effects of two antihypertensive therapies. The participants were divided into quartiles of baseline serum total adiponectin or high molecular weight (HMW) adiponectin. Multivariable Cox proportional hazards analysis was performed to determine the prognostic factors associated with CV events. Kaplan-Meier analysis for CV events by quartiles of baseline total adiponectin showed that patients in the highest total adiponectin quartile (Q4) had more CV events (P = 0.0135). On the other hand, no significant difference was noted regarding the incidence of CV events among patients stratified by HMW adiponectin quartile (P = 0.2551). Even after adjustment for potential confounders, the highest total adiponectin quartile (Q4) remained independently associated with incident CV events in hypertensive patients (HR = 1.949: 95%CI 1.051–3.612; P = 0.0341). These results showed that total adiponectin, but not HMW adiponectin, was independently associated with the incidence of CV events in treated hypertensive patients, thereby highlighting total adiponectin as a valuable predictor for hypertensive cardiovascular outcomes.

## Introduction

Adiponectin is the most abundant serum adipocytokine primarily secreted from adipose tissue and its serum concentrations decrease with obesity^1–3^. *In vivo* and *in vitro* experimental studies^2–4^ indicate that adiponectin exerts pleiotropic beneficial effects such as insulin-sensitizing, anti-inflammatory, anti-atherosclerotic, and anti cardioprotective effects. Low plasma adiponectin levels in humans are an indicator of the metabolic syndrome and associated with unfavourable cardiovascular and metabolic risk profile^1,3,5,6^, including insulin resistance, type 2 diabetes, low HDL-cholesterol, etc. Furthermore, low plasma adiponectin levels are significantly related to high risk of coronary artery disease in men^7^, an increased risk of mortality after ischemic stroke^8^, and neurological severity in ischemic stroke patients^9^. High plasma adiponectin concentrations are associated with lower risk of myocardial infarction in men^10^ and are associated with a reduced risk for incident coronary heart disease events among men with type 2 diabetes^11^. Moreover, the meta-analysis also showed that higher adiponectin levels were associated with a low risk of coronary heart disease^12^. In contrast to the abundant evidence indicating inverse association of adiponectin levels in adverse cardiovascular outcomes, multiple lines of studies show conflicting findings proposing “adiponectin paradox” and indicate that high adiponectin concentrations are associated with adverse cardiovascular outcomes in various populations, such as patients with ischemic heart disease^13,14^, acute coronary syndrome (ACS)^15,16^, chronic heart failure^17–19^, prevalent CVD^20^, chronic kidney disease^21^, a relative young multi-ethnic population cohort^22^, or older adults^23^. Thus, the previous investigations show controversial findings regarding the association of adiponectin levels with cardiovascular outcomes.

In spite of the abundant reports on the association of adiponectin levels with various populations, little is known about the clinical value of circulating adiponectin levels in the pathophysiology of hypertension^24–30^. Most importantly, the association of adiponectin levels with CV outcomes in hypertensive patients remains to be determined. ATTEMPT-CVD study^31,32^ is a multicenter, prospective, randomized study to investigate the comparative effects of angiotensin receptor blocker (ARB)-based antihypertensive therapy versus non-ARB-based antihypertensive therapy on the change in various biomarkers and incident composite CV events in hypertensive outpatients with at least one cardiovascular risk. The prespecified biomarkers as the endpoint of ATTEMPT-CVD study included serum total adiponectin and serum high molecular weight (HMW) adiponectin^31,32^. Therefore, in the present study, to investigate the prognostic value of baseline serum total adiponectin and HMW adiponectin in hypertensive patients, we performed subanalysis on 1,228 hypertensive patients enrolled in the ATTEMMPT-CVD study.

## Results

### Baseline characteristics of patients categorized by quartile of serum total adiponectin

In the present study, serum total adiponectin levels at baseline were available for 1,228 patients enrolled in the ATTEMPT-CVD study. Table 1 shows baseline characteristics of patients categorized by quartile of serum total adiponectin at baseline. The median (25th-75th percentile) total adiponectin concentration was 5.28 (3.56–7.87) μg/mL. Patients with the highest total adiponectin levels in Q4 versus Q1, Q2, or Q3 were older (P < 0.001), were less likely to be male (P < 0.001), had lower BMI (P = 0.002), had lower diastolic BP(P < 0.001), were less likely to be current smoker (P < 0.001), while, there was no difference among 4 groups of total adiponectin quartile with respect to systolic BP, proportion of diabetes mellitus, proportion of hyperlipidemia, proportion of previous cardiovascular disease, proportion of allocation to ARB based antihypertensive therapy.

**Table 1:** Baseline characteristics of patients categorized by quartile of serum total adiponectin.

	Quartile of serum total adiponectin (Total adiponectin level (μg/mL))				
	Q1 (0.84–3.56)	Q2 (3.57–5.28)	Q3 (5.29–7.87)	Q4 (7.88–41.59)	P value
Number in each quartile	308	308	306	306	
Age (years)	62.7 ± 9.5**	64.7 ± 9.9**	67.6 ± 8.6**	70.2 ± 7.7	<0.001
Male, n (%)	248 (81)	194 (63)	153 (50)	120 (39)	<0.001
BMI (kg/m^2^)	25.6 ± 3.4**	25.6 ± 3.9*	25.1 ± 3.8	24.7 ± 4.2	0.002
Systolic BP (mmHg)	151.4 ± 16.6	150.5 ± 15.7	150.0 ± 14.5	149.9 ± 16.0	0.872
Diastolic BP (mmHg)	86.7 ± 11.4**	85.1 ± 11.9**	83.7 ± 11.1*	81.0 ± 12.0	<0.001
Heart rate (b.p.m)	72.9 ± 11.0	71.7 ± 10.0	71.6 ± 11.6	72.6 ± 11.2	0.508
Diabetes mellitus, n (%)	218 (71)	191 (62)	199 (65)	211 (69)	0.103
Hyperlipidemia, n (%)	193 (63)	178 (58)	167 (55)	166 (54)	0.122
Current smoker, n (%)	80 (26)	61 (20)	42 (14)	33 (11)	<0.001
Previous cardiovascular disease, n (%)	158 (51.3)	176 (57.1)	183 (59.8)	181 (59.2)	0.131
Allocation to ARB therapy, n (%)	142 (46.1)	162 (52.6)	160 (52.3)	151 (49.3)	0.335
HMW adiponectin (μg/mL)	0.86** (0.62–1.14)	1.84** (1.57–2.16)	3.32** (2.75–3.87)	6.44 (5.24–8.83)	<0.001
HMW/total adiponectin ratio	0.31 ± 0.11**	0.43 ± 0.09**	0.51 ± 0.09**	0.62 ± 0.09	<0.001
Plasma BNP (pg/mL)	12.2** (6.2–21.9)	16.6** (8.2–28.7)	18.8** (9.3–37.7)	31.2 (16.9–61.0)	<0.001
UACR (mg/g creatinine)	24.7 (11.5–71.7)	25.5 (10.6–88.3)	22.3 (10.8–98.7)	33.8 (11.7–105.8)	0.125
eGFR (ml/min per 1.73 m^2^)	75.1** (65.0–88.7)	72.2** (61.7–85.7)	68.5 (57.2–83.8)	67.5 (55.8–78.8)	<0.001
hsCRP (ng/mL)	793** (413–1838)	637** (348–1188)	561** (283–1258)	400 (178–892)	<0.001
Urinary 8-OHdG(ng/mL)	11.0** (6.7–17.4)	9.9 (5.8–14.6)	9.1 (5.2–14.5)	8.8 (5.1–14.2)	0.001
Serum or plasma values					
Creatinine (mg/dL)	0.79 ± 0.20	0.78 ± 0.23	0.79 ± 0.25	0.79 ± 0.26	0.570
Potassium (mEq/L)	4.26 ± 0.48*	4.27 ± 0.55	4.30 ± 0.46	4.37 ± 0.58	0.029
Total cholesterol (mg/dL)	197 ± 36	197 ± 40	196 ± 34	194 ± 33	0.677
LDL cholesterol (mg/dL)	116 ± 31**	114 ± 30*	112 ± 28*	107 ± 29	<0.001
HDL cholesterol (mg/dL)	51 ± 11**	55 ± 12**	56 ± 13**	62 ± 15	<0.001
Blood sugar (mg/dL)	144 ± 62*	132 ± 52	131 ± 53	132 ± 56	0.015
Hemoglobin A1c (%)	6.6 ± 1.3**	6.3 ± 1.2	6.3 ± 1.1	6.3 ± 1.1	0.001
Uric acid (mg/dL)	5.6 ± 1.3**	5.4 ± 1.4**	5.2 ± 1.3	5.1 ± 1.3	<0.001

Abbreviations: BMI, body mass index; BP, blood pressure; ARB, antihypertensive treatment with angiotensin II receptor blocker; HMW, high molecular weight; BNP, brain natriuretic peptide; UACR, urinary albumin/creatinine ratio; eGFR, estimated glomerular filtration rate; hsCRP, high sensitive C-reactive protein; 8-OHdG, 8-hydroxy-2′-deoxyguanosine; LDL, low-density lipoprotein; HDL, high-density lipoprotein. HMW adiponectin, plasma BNP, UACR, eGFR, hsCRP and urinary 8-OHdG are expressed as median with interquartile range. Other data are mean ± s.d. for continuous values and number (%) for categorical variables. P-value was calculated using Steel-Dwass or Tukey’s multiple comparison test for continuous variables and χ^2^ test for categorical variables. *P < 0.05, **p < 0.01 vs Q4.

Patients with total adiponectin levels in Q4 versus Q1, Q2, or Q3 had higher serum HMW adiponectin (P < 0.001), higher HMW/total adiponectin ratio (P < 0.001), higher plasma BNP (P < 0.001), lower eGFR(P < 0.001), lower hsCRP (P < 0.001), lower urinary 8-OHdG (P = 0.001), lower LDL-cholesterol (P < 0.001), higher HDL-cholesterol(P < 0.001), lower blood sugar (P = 0.015), lower hemoglobin A1c (P = 0.001), and lower uric acid (P < 0.001). While, there was no significant difference among patients with total adiponectin quartiles regarding UACR (P = 0.125).

### Correlation of total adiponectin with other biomarkers

Supplementary Table 1 shows Spearman correlation coefficients between baseline serum total adiponectin and other biomarkers. Continuous serum total adiponectin concertation at baseline showed strong positive correlation with serum HMW adiponectin (r = 0.9756, P < 0.0001) and with HMW /total adiponectin ratio (r = 0.8195, P < 0.0001). Serum total adiponectin exhibited weak positive correlation with age (r = 0.3292, P < 0.0001) and plasma BNP (r = 0.3491, P < 0.0001), while exhibited very weak negative correlation with BMI (r = −0.1107, P = 0.0001), eGFR (r = −0.1817, P < 0.0001), serum hsCRP (r = −0.2179, P < 0.0001), urinary 8-OHdG(r = −0.1124, P < 0.0001), HbA1c (r = −0.07813, P = 0.0062), LDL-cholesterol (r = −0.1156, P < 0.0001), and uric acid (r = −0.1706, P < 0.0001). There was no correlation of serum total adiponectin with UACR (P = 0.0541) or systolic BP (P = 0.3056).

### Incidence of composite cardiovascular and renal events according to quartiles of serum total adiponectin

**Figure 1:**
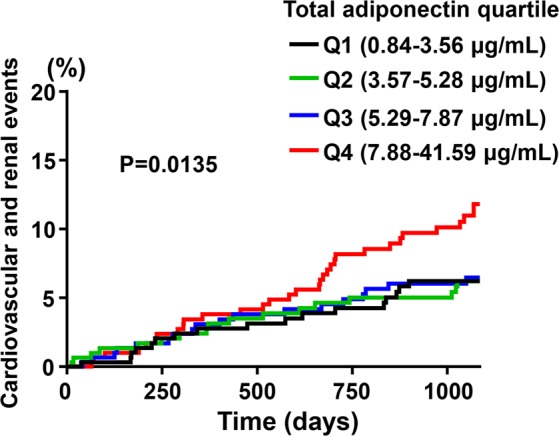
Kaplan-Meier curves for composite cardiovascular and renal events stratified by quartiles of serum total adiponectin at baseline. The number of occurrence of endpoints was 17, 18, 19, and 35 in Q1 (n = 306), Q2 (n = 302), Q3 (n = 303), and Q4 (n = 302), respectively.

Figure 1 shows Kaplan-Meier curves for composite cardiovascular and renal events in patients by serum total adiponectin quartile. There was the significant difference in incident cardiovascular and renal events among total adiponectin quartiles (P = 0.0135) and patients with total adiponectin concentration in Q4 had more cardiovascular and renal events. Detail of composite cardiovascular and renal events among patients in Q1-Q4 was shown in Table 2.

**Table 2:** Detail of composite cardiovascular and renal events among patients categorized by quartile of serum total adiponectin.

Cardiovascular and renal events	Quartile of total adiponectin			
	Q1	Q2	Q3	Q4
	(n = 306)	(n = 302)	(n = 303)	(n = 302)
Total, n	17	18	19	35
Stroke, n	5	2	5	7
Transient ischemic attack, n	1	1	1	0
Sudden death, n	1	0	1	2
Acute myocardial infarction, n	1	1	2	2
Angina pectoris, n	0	1	2	3
Heart failure, n	0	2	1	6
Aortic aneurysm, n	2	0	0	1
Aortic dissection, n	0	1	0	0
Peripheral artery disease, n	3	2	2	3
Diabetic nephropathy, n	1	1	0	0
Diabetic retinopathy, n	3	2	3	5
Doubling of serum creatinine, n	0	5	1	6
End stage renal disease, n	0	0	1	0

### Blood pressure of patients with total adiponectin quartile during follow up period

During 3 years of follow up period, systolic BP was similar among total adiponectin quartiles (Supplementary Table 2). Although diastolic BP of patients with Q4 was slightly lower among total adiponectin quartiles during the follow up period, the difference was very small.

### Incidence of composite cardiovascular and renal events according to quartile of serum HMW adiponectin or HMW/total adiponectin ratio

**Figure 2:**
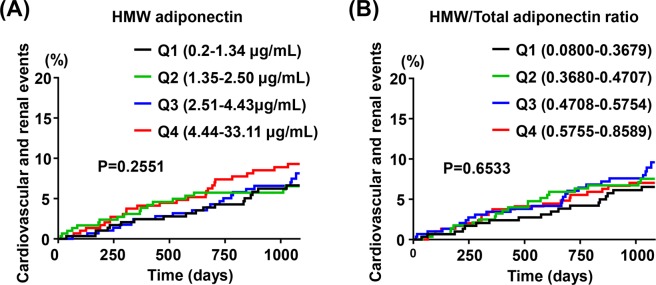
Kaplan-Meier curves for composite cardiovascular and renal events stratified by quartiles of serum HMW adiponectin (**A**) or by quartiles of HMW/total adiponectin ratio (**B**). (**A**) The number of occurrence of endpoints was 18, 19, 22, and 30 in Q1 (n = 306), Q2 (n = 303), Q3 (n = 301), and Q4 (n = 303), respectively. (**B**) The number of occurrence of endpoints was 18, 21, 25, and 25 in Q1 (n = 306), Q2 (n = 303), Q3 (n = 300), and Q4 (n = 304), respectively.

Differing from the case of total adiponectin, there was no significant difference in incidence of composite cardiovascular and renal events among patients with quartile of HMW adiponectin (P = 0.2551) (Fig. 2(A)) or HMW/total adiponectin ratio (P = 0.6533)(Fig. 2(B)).

### Association of prognostic factors with composite cardiovascular and renal events

Table 3 shows results of the multivariable Cox regression analysis with the backward selection method to determine an adjusted hazard ratio of prognostic factor for composite cardiovascular and renal events. The original model included the following 11 covariates: total adiponectin Q2, total adiponectin Q3, total adiponectin Q4, sex, age, presence of previous cardiovascular diseases, presence of previous diabetes mellitus, UACR, plasma BNP, eGFR, and current smoking. Total adiponectin Q1 was used as the reference value (hazard ratio = 1). In this analysis, age (P = 0.9692), eGFR (P = 0.0942), and current smoking (P = 0.0628) were not significantly associated with composite cardiovascular and renal events in overall patients, and hence, these covariates were deleted. On the other hand, total adiponectin concentration in Q4 was independently associated with composite cardiovascular and renal events (HR = 1.949: 95%CI 1.051–3.612; P = 0.0341), while total adiponectin in Q2 (P = 0.6337) or Q3 (P = 0.7518) had no significant association with cardiovascular and renal events. Male gender (P = 0.009), previous cardiovascular disease (P = 0.0002), previous type 2 diabetes (P < 0.0001), UACR ≥ 30 mg/g creatinine (P = 0.0005), and plasma BNP ≥ 19 mg/ml (P = 0.0053) were also significantly associated with cardiovascular and renal events.

**Table 3:** Adjusted hazard ratios of prognostic factor for composite cardiovascular and renal events in overall patients by multivariable Cox proportional analysis with the backward selection method.

	HR (95%CI)	P-value
Total adiponectin Q1 (Reference)	1	
Total adiponectin Q2	1.177 (0.602–2.302)	0.6337
Total adiponectin Q3	1.114 (0.570–2.177)	0.7518
Total adiponectin Q4	1.949 (1.051–3.612)	0.0341
Male gender	1.851 (1.166–2.936)	0.0090
Previous cardiovascular disease	2.521 (1.541–4.123)	0.0002
Previous type 2 diabetes	3.077 (1.762–5.375)	<0.0001
UACR ≥ 30 mg/g creatinine	2.290 (1.438–3.645)	0.0005
Plasma BNP ≥ 19 mg/ml	1.921 (1.214–3.040)	0.0053

The original model included the following 11 covariates: Total adiponectin Q2, Total adiponectin Q3, Total adiponectin Q4, sex, age, presence of baseline cardiovascular diseases, presence of baseline type 2 diabetes, UACR, plasma BNP, eGFR, and current smoking. Of the 11 covariates, age (P = 0.9692), eGFR (P = 0.0942), and current smoking (P = 0.0628) had no significant association with composite cardiovascular and renal events in overall patients, and therefore these covariates were deleted. Abbreviations: HR, hazard ratio; 95%CI, 95% confidence interval; UACR, urinary albumin/creatinine ratio; BNP, brain natriuretic peptide. Total adiponectin concentration in Q1 was used as the reference (HR = 1.0).

## Discussion

To our knowledge, the association of circulating adiponectin levels with cardiovascular and renal outcomes in hypertensive patients remains unknown. The major findings of our present study were that high total adiponectin was independently associated with incident cardiovascular and renal events in hypertensive patients receiving antihypertensive therapies and that in contrast to total adiponectin, HMW adiponectin had no significant association with cardiovascular and renal events. Thus, our findings suggest that total adiponectin, but not HMW adiponectin, may be a valuable predictor for incident cardiovascular and renal events in hypertensive patients.

Preclinical study shows that adiponectin-knockout mice exhibit the elevation of blood pressure and adiponectin supplementation significantly lowers blood pressure in hypertensive obese mice^33^. Therefore, adiponectin seems to play a protective role against hypertension. However, in clinical settings, the previous findings regarding the association of total adiponectin in the risk of hypertension are controversial^24–30^. Prospective studies in Chinese^25^ and Japanese^26^ populations and a systematic review and meta-analysis^27^ show that hypoadiponectinemia is significantly associated with high risk of hypertension. High total adiponectin levels are associated with reduced risk of incident hypertension^29^. On the other hand, other studies^24,28,30^ showed no association of total adiponectin with risk of hypertension. Thus, the predictive value of adiponectin in the incidence of hypertension remains to be defined. Furthermore, all previous studies^24–30^ focused on the association of adiponectin only with incidence of hypertension, and have not examined the association with the incidence of hypertensive adverse cardiovascular outcomes. Thus, at present, the association of adiponectin levels with cardiovascular and renal events in hypertensive patients is unknown. These findings encouraged us to investigate the association of baseline adiponectin levels with cardiovascular and renal events among hypertensive patients enrolled in the ATTEMPT-CVD study.

It is an important issue whether potential difference in BP among quartiles of total adiponectin might contribute to the higher incidence of cardiovascular and renal events in the highest total adiponectin quartile (Q4), since ATTEMPT-CVD study was designed to compare the effect of two antihypertensive therapies and adiponectin provokes antihypertensive effect evidenced by animal study^33^. However, in the present analysis, systolic BP at baseline and during antihypertensive treatment was comparable among patients stratified by quartile of total adiponectin. Moreover, patients in the highest total adiponectin quartile (Q4) had slightly lower diastolic BP at baseline and during antihypertensive treatment, although the difference was very small. Thus, BP seems not to be related to higher incidence of cardiovascular and renal events in the highest total adiponectin quartile (Q4).

Circulating adiponectin levels are known to be affected by various factors, such as plasma BNP, renal function, aging and sex^2,34–36^. In the present study, increasing total adiponectin from Q1 to Q4 was associated with the increased age, more female proportion, and increased plasma BNP, while associated with the decreased eGFR. In the present study, we have carried out multivariable Cox proportional hazards analysis with the backward selection starting with 11 covariates including age, sex, plasma BNP, and eGFR, etc. Of note, even after adjustment for potential confounders, the association of the highest total adiponectin quartile (Q4) with composite cardiovascular and renal events remained significant. Therefore, our study showed that high total adiponectin is independently associated with the incidence of cardiovascular and renal events in hypertensive patients.

Adiponectin in human serum exists as a trimer, a hexamer, and HMW form^34,37^. Importantly, HMW adiponectin is regarded to be the most active form, and is more associated with insulin sensitivity and with lower risk for incident diabetes than total adiponectin^38,39^, and confers more protection against endothelial cell apoptosis^40^. Based on these findings, it has been proposed that HMW adiponectin concentrations and HMW/total adiponectin ratio^37,41^ may be more useful biomarker than total adiponectin for evaluating risk of cardiovascular and renal diseases. However, almost previous studies regarding the association of adiponectin with cardiovascular outcomes among various populations have been limited to total adiponectin. Limited numbers of studies regarding HMW adiponectin have been reported and showed inconsistent findings on the association with cardiovascular outcomes^19,37,38,42–44^. Moreover, the association of HMW adiponectin or HMW/total adiponectin ratio with hypertensive cardiovascular events has not been reported. In the present study, we found strong positive correlation of total adiponectin with HMW adiponectin and HMW/total adiponectin ratio. However, in contrast to the significant association of total adiponectin with cardiovascular and renal events, neither HMW adiponectin nor HMW /total adiponectin ratio was associated with the incidence of cardiovascular and renal events in hypertensive patients. Therefore, our present findings suggest that total adiponectin may be more useful predictor for hypertensive cardiovascular and renal events than HMW adiponectin.

The present study did not allow us to elucidate the mechanism and the significance of high circulating adiponectin levels in the Q4 group of hypertensive patients. It has been proposed that high circulating adiponectin levels in various populations such as heart failure, ischemic heart disease or CKD etc. might be attributed to counter-regulatory upregulation of adiponectin production in response to various stresses caused by such severe chronic diseases^2,13–15,17,21^. Accumulating experimental evidence indicates that adiponectin has pleiotrophic beneficial effects including anti-inflammatory, antiatherogenic, or cardioprotective actions^3,45–47^. Therefore, it is likely that high circulating adiponectin levels in the Q4 group of high-risk hypertensive patients might be at least partially explained by compensatory upregulation of adiponectin production in response to severe chronic stress related to hypertension. However, animal and clinical studies show that functional adiponectin resistance develops in various chronic diseases such as obesity, diabetes mellitus or heart failure^2,45,48–51^. Previous reports^50,51^ show that the increased circulating adiponectin levels in patients with heart failure are accompanied by the downregulation of its adiponectin receptor and decreased downstream signaling such as deactivation of the PPAR-α/AMPK pathway and downregulation of several target genes in skeletal muscles, thereby proposing the failure of adiponectin to exert significant beneficial effects (functional adiponectin resistance) in such chronic disease. Therefore, it remains uncertain whether high adiponectin levels in Q4 quartile might significantly exert cardiovascular protective effect. Alternatively, *in vitro* study^52^ indicates that adiponectin binds C1q and activates the classical pathway of complement, thereby suggesting that adiponectin may exert pro-inflammatory action rather than anti-inflammatory action. Therefore, it cannot be completely excluded that high adiponectin levels in the Q4 group might be involved in the progression of hypertensive CV events through pro-inflammatory action^2,52^. Further study is required to elucidate the mechanism for such compensatory response of adiponectin and its protective or detrimental role in cardiovascular diseases in the clinical setting.

### Study limitation

There are several limitations in this study. First, the present study was a post-hoc analysis of ATTEMPT-CVD study, although serum total adiponectin and HMW adiponectin were prespecified as a secondary endpoint of the ATTEMPT-CVD study. Second, the present study did not allow us to elucidate the causal relationship between high total adiponectin and the increased cardiovascular and renal events in hypertensive patients. Third, in the present study, increasing total adiponectin levels from Q1 to Q4 led to beneficial cardiovascular profile as shown by the decrease in diastolic BP, inflammatory marker (hs-CRP), oxidative stress marker (8-OHdG), HbA1c, blood sugar, and LDL-cholesterol and the increase in HDL-cholesterol. It is unclear whether such beneficial cardiovascular profile in high total adiponectin quartile might be attributed to compensatory mechanism mediated by pleiotrophic effects of adiponectin, although the beneficial effects of adiponectin shown by experimental studies might be partially involved in such beneficial cardiovascular profile. Fourth, it cannot be completely excluded that unknown confounding factor(s) might affect our present findings, although the association of total adiponectin with cardiovascular and renal events remained significant even after adjustment for potential confounders. Fifth, although circulating adiponectin derives primarily from adipose tissue, it is also produced by other types of cells including cardiomyocytes, skeletal muscles and endothelial cells. However, it remains uncertain whether adiponectin from non-adipose tissue significantly contributed to high adiponectin levels in Q4 quartile. Finally, the underlying mechanism of differential impacts of total and HMW adiponectin on cardiovascular outcomes in hypertension is not elucidated in this study.

In conclusion, we obtained the evidence that total adiponectin, but not HMW adiponectin, was independently associated with the incidence of cardiovascular and renal events in hypertensive patients under antihypertensive therapies. Thus, we propose that total adiponectin may be a useful predictor for incident cardiovascular and renal events in hypertensive patients. However, larger scale prospective study is required to define the precise value of total adiponectin and HMW adiponectin as a predictive marker for hypertensive cardiovascular and renal outcomes.

## Patients and Methods

### The inclusion and exclusion criteria, and study protocol of ATTEMPT-CVD

The study design^32^ and the primary results^31^ of ATTEMPT-CVD study (ClinicalTrials. gov number NCT01075698) have been previously published.

The ATTEMPT-CVD study was a multicenter, prospective, randomized open label, controlled trial to compare the effect of an angiotensin receptor blocker (ARB)(telmisartan)-based antihypertensive therapy vs non-ARB antihypertensive therapy on various biomarkers and composite cardiovascular and renal events in hypertensive outpatients^31,32^. Patients were followed up for three years at 168 institutions throughout Japan. Patients were eligible if they were 40–79-year-old hypertensive outpatients who had at least one cardiovascular risk (type 2 diabetes, renal factors, cardiac factors, cerebral factors and peripheral arterial factors). The exclusion criteria included type 1 diabetes, severe renal disorder (serum creatinine level ≥2.0 mg/dL) and the occurrence of heart failure NYHA Classification III or IV, myocardial infarction, percutaneous revascularization and bypass grafting of coronary artery/lower extremity vessel, cerebral infarction, cerebral hemorrhage, subarachnoid hemorrhage and transient cerebral ischemic attack within 6 months before the observation period, and it also included malignant hypertension, secondary hypertension, pregnant women, clinically problematic allergic disease or past history of hypersensitivity to the drugs used, past history of significant adverse drug reactions, extremely poor biliary secretion or serious hepatic disorder, patients who require treatment for a malignant tumor, and other patients who are judged by the physician to be unsuitable for the study. Full inclusion and exclusion criteria are described in our protocol paper^32^. All participants provided written informed consent. The study protocol is in agreement with the guidelines of the ethics committees at our institutions and the study complies with the Declaration of Helsinki. The institutional review board of each participating hospital approved this trial (See Appendix A), and written informed consent was obtained from each patient. The study protocol was approved by Independent Ethics Committee of Kumamoto University.

The physicians examined the survey items at the start of the study (at registration), after 3, 6, 12, 24 and 36 months from the start of the study, at a discontinuation/dropout, at the occurrence of any cardiovascular and renal event, and at the occurrence of any adverse event.

### Endpoints of ATTEMPT-CVD study

In ATTEMPT-CVD study, the primary endpoints were changes in urinary albumin/creatinine ratio (UACR) and changes in plasma BNP levels from baseline, as described. The incidence of composite cardiovascular and renal events was a prespecified secondary endpoint of ATTMEPT-CVD study. The composite cardiovascular and renal events included cerebral events (cerebral infarction, cerebral hemorrhage, subarachnoid hemorrhage, unknown type of stroke, transient ischemic attack), cardiac events (sudden death, myocardial infarction, angina pectoris, asymptomatic myocardial ischemia, heart failure), aortic/peripheral arterial events (aortic aneurysm, aortic dissection, arteriosclerotic disease), newly occurred (or) aggravated diabetic complications (diabetic nephropathy, diabetic retinopathy, diabetic neuropathy), aggravation of renal function (doubling of serum creatinine level, initiation of dialysis, renal transplantation).

In addition, the changes in serum total adiponectin, serum high molecular weight (HMW) adiponectin, plasma high sensitive C-reactive protein (hsCRP), urinary 8-hydroxy-2′-deoxyguanosine (8-OHdG), and estimated glomerular filtration rate (eGFR) were also specified as the secondary endpoints of ATTEMPT-CVD study.

### Measurement of adiponectin and other biomarkers

The plasma or serum concentrations of all biomarkers were measured in SRL, Inc. (Tokyo, Japan). Serum total adiponectin and HMW adiponectin concentrations were measured by ELISA kit (Sekisui Medical Co., LTD., Tokyo, Japan)

### Statistical analyses

All analyses were performed on the intention-to-treat population. Serum total adiponectin concentration, HMW adiponectin concentration, and HMW/total adiponectin ratio at baseline were categorized by quartiles (Q1, Q2, Q3, Q4). As for cardiovascular and renal events, time to first event curves were estimated by the Kaplan-Meier method and the log-rank test was used to analyze the differences among patients with quartiles (Q1, Q2, Q3, Q4) of serum total adiponectin, HMW adiponectin, or HMW/total adiponectin ratio. Using Cox proportional hazard model, the hazard ratio (HR) of Q1–Q4 and its 95% confidence interval (CI) were calculated. Two-way repeated measures analysis of variance was used to compare among Q1–Q4 for time course of systolic or diastolic blood pressure during the follow-up period. Multivariable Cox proportional hazards analysis combined with the backward selection method was performed to determine the association of total adiponectin with the incidence of composite cardiovascular and renal events independently. The original model included the following 11 covariates: total adiponectin in Q2, Q3, and Q4, sex, age, presence of baseline cardiovascular diseases, presence of baseline type 2 diabetes, eGFR, plasma BNP, UACR, and current smoking. Total adiponectin in Q1 was used as the reference value and its HR was expressed as 1. To compare baseline parameters among patients in Q1–Q4, P-value was calculated using Steel-Dwass or Tukey’s multiple comparison test for continuous variables and χ^2^ test for categorical variables. Spearman correlation coefficients were calculated between total adiponectin concentration and others biomarkers at baseline. Windows SAS Version 9.2 and subsequent versions were used as the statistical analysis software. P-values of less than 0.05 were considered statistically significant.

## Supplementary information

Supplementary Information

## Data Availability

The datasets generated during and/or analysed during the current study are available from the corresponding author on reasonable request.
