# Supplementary material for: Total adiponectin is associated with incident cardiovascular and renal events in treated hypertensive patients: subanalysis of the ATTEMPT-CVD randomized trial
Source: Sci Rep. 2019 Nov 12;9:16589. doi: 10.1038/s41598-019-52977-x (PMC6851137; doi:10.1038/s41598-019-52977-x)
Supplement: Supplementary file 1 — Supplementary Information [file 41598_2019_52977_MOESM1_ESM.pdf]

Supplementary Information

Total adiponectin is associated with incident cardiovascular and renal events in treated hypertensive patients: subanalysis of the ATTEMPT-CVD randomized trial

Shokei Kim-Mitsuyama*[1], Hirofumi Soejima[2,3], Osamu Yasuda[4], Koichi Node[5], Hideaki Jinnouchi[6], Eiichiro Yamamoto[2], Taiji Sekigami[7], Hisao Ogawa[8], Kunihiko Matsui[9]

[1]Department of Pharmacology and Molecular Therapeutics, Graduate School of Medical Sciences, Kumamoto University, Kumamoto, Japan; [2]Department of Cardiovascular Medicine, Graduate School of Medical Sciences, Kumamoto University, Kumamoto, Japan; [3]Health Care Center, Kumamoto University, Kumamoto, Japan; [4]Department of Sports and Life Sciences, National Institute of Fitness and Sports in Kanoya, Kanoya, Japan; [5]Department of Cardiovascular Medicine, Saga University, Saga, Japan; [6]Diabetes Care Center, Jinnouchi Clinic, Kumamoto, Japan; [7]Division of Internal Medicine & Diabetes and Endocrine, Sekigami Clinic, Yatsushiro, Japan; [8]National Cerebral and Cardiovascular Center, Suita, Japan; [9]Department of General and Community Medicine, Kumamoto University Hospital, Kumamoto, Japan

All correspondence to: Shokei Kim-Mitsuyama, MD., PhD, FAHA, FESC

Department of Pharmacology and Molecular Therapeutics, Kumamoto University Graduate School of Medical Sciences 1-1-1 Honjyo, Kumamoto 860-8556, Japan

Tel. 81-96-373-5082 Fax. 81-96-373-5082

E-mail: mitsuyam@gpo.kumamoto-u.ac.jp

Supplementary Information

Supplementary Table 1. Spearman correlation coefficients between baseline total adiponectin and other biomarkers

	r	p
Age	0.3292	< 0.0001
BMI (kg/m^2)	-0.1107	0.0001
SBP (mmHg)	-0.02926	0.3056
HMW adiponectin (μg/mL)	0.9756	< 0.0001
HMW/total adiponectin ratio	0.8195	< 0.0001
Plasma BNP (pg/mL)	0.3491	< 0.0001
UACR (mg/g creatinine)	0.05498	0.0541
eGFR	-0.1817	< 0.0001
Serum hsCRP (ng/mL)	-0.2179	< 0.0001
Urinary 8-OHdG (ng/mL)	-0.1124	< 0.0001
HbA1c (%)	-0.07813	0.0062
LDL-cholesterol (mg/dL)	-0.1156	< 0.0001
Uric acid (mg/dL)	-0.1706	< 0.0001

Abbreviations: BMI, body mass index; SBP, systolic blood pressure; HMW, high molecular weight; BNP, brain natriuretic peptide; UACR, urinary albumin/creatinine ratio; eGFR, estimated glomerular filtration rate; hsCRP, high sensitive C-reactive protein; 8-OHdG, 8-hydroxy-2'-deoxyguanosine; HbA1c, hemoglobin A1c; LDL, low-density lipoprotein.

Supplementary Information

Supplementary Table 2. Time course of systolic and diastolic BP of patients categorized by quartile of serum total adiponectin

	0 months	3 months	6 months	12 months	24 months	36 months	P value*
Systolic BP (mmHg)							0.6637
Q1	151.4±16.6	134.0±14.4	133.5±14.2	133.6±13.8	134.0±13.2	131.5±13.4	
Q2	150.5±15.7	134.7±15.1	132.7±13.9	133.0±13.6	132.2±13.8	131.7±16.3	
Q3	150.0±14.5	134.2±13.3	132.4±12.7	132.8±13.2	130.5±13.2	130.7±15.4	
Q4	149.9±16.0	134.3±14.8	133.0±14.7	132.9±14.2	133.0±13.9	132.0±15.6	
Diastolic BP (mmHg)							<0.0001
Q1	86.7±11.4	78.7±10.0	78.5±9.6	78.5±9.8	77.5±10.2	75.9±9.4	
Q2	85.1±11.9	76.8±10.7	76.5±10.5	76.4±9.6	74.8±9.6	74.1±10.5	
Q3	83.7±11.1	76.0±10.6	75.2±10.1	75.0±10.2	73.6±10.3	73.3±10.6	
Q4	81.0±12.0	73.1±10.0	73.6±10.7	72.8±10.6	72.9±9.9	71.3±10.6	

Abbreviation: BP, blood pressure. *Two-way repeated measures analysis of variance was used to compare among Q1-Q4 for time course of systolic or diastolic blood pressure during the follow-up period. Values are mean±SD.

Supplementary Information

Appendix A

Ethics committees/institutional review boards of participating hospitals that approved this trial are:

山本クリニック，しもの循環器内科クリニック，伊勢内科医院，森田医院，林田クリニック，佐藤内科小児科取上医院，松園第二病院，本荘第一病院，待井循環器科内科クリニック，わかまつ内科クリニック，やまき内科クリニック，小金原診療所，児玉メディカルクリニック，廻田クリニック，馬場医院，緑風荘病院，安部医院，おおり医院，やまがみ内科医院，横須賀市立うわまち病院，鶴見中央クリニック，山本クリニック，わかさ内科クリニック，木村病院，中島医院，おがわ内科クリニック，田中内科医院，内山医院，稲沢市民病院，静岡県立静岡病院，草津総合病院，南港クリニック，大阪府済生会野江病院，大阪府済生会千里病院，きぬがわ内科循環器内科，秋岡診療所，はやし内科クリニック，勝谷医院，東宝塚さとう病院，守山市民病院，かとう内科並木通り診療所，重信医院，中洲八木病院，ホウエツ病院，美波町国民健康保険由岐病院，小松内科循環器科クリニック，愛媛県立新居浜病院，花岡内科循環器科医院，乙成内科医院，大牟田天領病院，福岡徳洲会病院，新行橋病院，香月内科クリニック，横田内科，みやた内科医院，佐々木医院，三嶋内科，串間市民病院，高千穂町国民健康保険病院，佐藤医院，神宮医院，杉本病院，伊万里市立市民病院，織田病院，済生会唐津病院，權藤医院，池田内科皮膚科医院，唐津赤十字病院，内藤医院北山診療所，佐賀大学医学部附属病院，白石共立病院，飯盛内科，長生堂渡辺医院，吉田病院，山元記念病院，志田病院，大町町立病院，

Supplementary Information

城内病院，野田好生医院，せとぐち内科，古賀内科，佐賀県立病院好生館，佐賀社会保険病院，ニコークリニック，おおしまクリニック，藤﨑医院，奥内科医院，垂水中央病院，田中脳神経外科クリニック，坂元内科クリニック，大嶋医院，新別府病院，菅原内科杵築，小路内科医院，上人病院，井野辺府内クリニック，南部徳洲会病院，菅海明堂医院，陣内病院，まつおクリニック，八代総合病院，坂梨ハートクリニック，金森医院，宮城循環器内科，小沢医院，深水医院，しばた内科胃腸科，芦北クリニック，坂本内科循環器科医院，三森循環器科・呼吸器科病院，小野主生医院，大森医院，十善病院，熊本市立植木病院，本庄内科病院，矢部広域病院，百崎内科医院，吉成外科内科医院，寺尾病院，問端内科，NTT 西日本九州病院，熊本大学医学部附属病院，土井医院，尾崎医院，くわみず病院，やまうち医院，球磨郡公立多良木病院，阿蘇立野病院，熊本市民病院，済生会熊本病院，原口循環器科内科医院，辻循環器科・内科，木山・中村クリニック，葦原医院，大林循環器科内科医院，直海内科クリニック，堀尾内科医院，岩井クリニック，小国公立病院，水前寺とうや病院，熊本機能病院　，東熊本病院，堤病院，あきた病院，荒尾中央病院，ニュー天草病院　，片岡内科医院，九州記念病院，球磨村診療所，沢田内科医院，青磁野リハビリテーション病院，東病院，はくざん胃腸科循環器科クリニック，ひとよし内科，堀田循環器内科，牧内科循環器科医院，佐々木脳神経外科，中邑医院，土井内科胃腸科医院，健康保険人吉総合病院，前田内科医院，大塚医院，宗像医院，西山医院，あけぼの第２クリニック，竜山内科リハビリテーション病院，花園内科クリニック，にしくまもと病院

Supplementary Information

Appendix B

A Trial of Telmisartan Prevention of Cardiovascular Diseases (ATTEMPT-CVD) Investigators

Steering Committee: Hisao Ogawa, Shokei Kim-Mitsuyama, Koichi Node, Hideaki Jinnouchi, and Taiji Sekigami

Committee on Validation of Data and Events: Sohichi Uekihara, Yoichiro Hashimoto, and Shuichi Oshima

Safety Monitoring Board: Kazuteru Fujimoto, Toshiro Yonehara, and, Teruyuki Hirano

Study Statistician: Kunihiko Matsui

Independent Data Management Center: Kaori Harada, Nobue Ueno, Yoshiko Terasaki, Mami Okamoto, Kanae Kanamori, Naoko Oka, Nami Matsuo, and, Kaori Sakaguchi

Investigators: Hirofumi Soejima, Osamu Yasuda, Megumi Yamamuro, Eiichiro Yamamoto, Yoichi Hanaoka, Hirofumi Kan, Tomio Jinnouchi, Syuichi Matsuo, Yutaka Wakasa, Naoto Yokota, Kazuo Matsunaga, Takatoshi Otonari, Toshihiko Sakanashi, Masanori Nishiyama, Hiroo Miyagi, Satoru Suzuki, Shukuko Ebihara, Masayasu Yamamoto, Ryo Fukami, Yoshimi Ohshima, Masahisa Ori, Junji Shibata, Daisuke Fujimatsu, Kouichi Mizobe, Michio Mizobe, Masato Nishimura, Shigeki Gondo, Juniti Miyata, Natsuki Nakamura, Koichi Kikuta, Yasuhiro Sakamoto, Shirou Mimori, Takashi Ono, Kunihiro Ohmori, Hirofumi Naitoh, Yasushi Suzuki, Shinichi Hirota, Kohji Honjo, Yoji Yamasaki, Kazushi Serikawa, Hideo Ikeda, Kazuo Ibaraki, Shigemasa Hashimoto, Sueo Momosaki, Satoshi Abe, Motoki Yoshinari, Kouji Sasaki, Masamitsu Toihata, Eiko Kobayashi, Takuo Ogawa, Shu Konno, Yuji Tanaka, Naoki

Yamamoto, Kazuya Shigenobu, Hiromi Fujii, Kazunori Murakami, Osamu Doi, Mari Kashiwabara, Kiyoharu Itoh, Fumihiro Oishi, Azusa Ikegami, Takao Mishima, Jouji Yamauchi, Shoichi Kitano, Hiroshi Haruguchi, Tomohiro Katsuya, Shunsuke Take, Sadao Nakajima, Haruaki Uchiyama, Yoshihiro Yamada, Munetaka Yamaguchi, Eiichiro Honda, Shinichi Uemura, Yasuhiro Morikami, Teruhiko Ito, Yoko Oe, Takashi Fukunaga, Yoshikuni Haraguchi, Masahiko Tsuji, Shigeru Kiyama, Kazuo Kuroki, Shigeya Tanaka, Kenichi Ashihara, Kenzo Motoki, Kenya Kusunose, Junko Hotchi, Hisashi Shimono, Kenichiro Iimori, Hisashi Watanabe, Fujio Yoshida, Kazuo Machii, Hiroshi Yamamoto, Kazuhisa Kodama, Atsushi Hiwatashi, Sumio Komatsu, Nakayasu Wake, Takashi Ise, Senshu Hifumi, Jun Ohbayashi, Shojiro Naomi, Yutaka Horio, Ken Iwai, Toshiro Matsunaga, Hiroshi Sakamoto, Yoshinobu Morikawa, Eisaku Harada, Yoshinobu Murasato, Masataka Horiuchi, Naomi Sugahara, Soichi Honda, Shingo Shoji, Masanori Shida, Toshinori Utsunomiya, Takao Baba, Moronari Matsunaga, Kazuyoshi Noda, Yoichi Setoguchi, Kohei Yamaguchi, Masayoshi Koga, Masashi Sakai, Yoshihisa Tamazaki, Genjiro Sato, Takanobu Nakajima, Masayuki Otani, Atsuyuki Wada, Jun Yamagami, Shinji Tamaki, Mitsuo Morita, Masako Waki, Tsukasa Katsuki, Kenji Sadamatsu, Yasuaki Koga, Shinsuke Tsuji, Yoshito Inobe, Kenji Misumi, Hiroto Okubo, Hirofumi Matsuda, Koichiro Kataoka, Kyoji Takaoka, Osamu Hashiguchi, Tomohiro Sawada, Tomonori Kanazawa, Kazuhiko Matsuo, Kunio Idegami, Eiichiro Tanaka, Yoshio Horita, Satoru Horita, Akira Maki, Koji Sasaki, Tomoki Nakamura, Masaru Doi, Katsunori Kawamitsu, Hirofumi Maeda, Takaoki Otsuka, Hiroyuki Tanaka, Takamasa Iwasawa, Kensei Hayashida, Hiroyuki Tanaka, Katuya Oosima, Yasuji Doi, Yasuhiro Nishiyama, Masashi Ikushima, Toru Kinugawa, Kaname Akioka, Yoko Anbe, Hiroaki Kono, Yasuhiro Sasaki, Mario Yamaki, Takashi Honda, Atsushi Sato, Takahiro

Supplementary Information

Hayashi, Yoshihiro Kimura, Aya Shiraki, Shunichi Sugimoto, Yasuharu Kodama, Hideki Wakamatsu, Shiho Enomoto, Yoshiyasu Yamatsu, Sadaaki Okamoto, Hiroaki Hosokawa, Kouji Maeno, Yohsuke Hanaoka, Hisanori Kanazawa, Tadasuke Chitose, Taishi Nakamura, Junichi Matsubara, Shinsuke Hanatani, Kenichi Tsujita, Satoshi Araki, Hiroaki Kusaka, Kenshi Yamanaga, Kensuke Toyama
